# In Vitro Antioxidant Activity of Green-Synthesized Zinc Oxide (ZnO) Nanoparticles Utilizing Extracts From Allium sativum

**DOI:** 10.7759/cureus.55184

**Published:** 2024-02-28

**Authors:** Benshiga E, Azhagu Madhavan Sivalingam, Arockia Alex, Brahma Neha

**Affiliations:** 1 Department of Community Medicine, Saveetha Medical College and Hospital, Saveetha Institute of Medical and Technical Sciences (SIMATS) Saveetha University, Chennai, IND; 2 Department of Biochemistry, Saveetha Medical College and Hospital, Saveetha Institute of Medical and Technical Sciences (SIMATS) Saveetha University, Chennai, IND

**Keywords:** antioxidant, sem, zno nanoparticle, characterization, allium sativum

## Abstract

Introduction: The primary objective of this study was to develop an environmentally friendly and efficient method for synthesizing zinc oxide (ZnO) nanoparticles (NPs), utilizing extracts from *Allium sativum* (garlic) plants, characterizing the synthesized ZnO NPs using various analytical techniques, and assessing their antibacterial and antioxidant properties.

Materials and Methods: The synthesis process involved utilizing extracts from garlic plants to create ZnO NPs. The NPs were subjected to comprehensive characterization through UV-visible (UV-vis) spectroscopy, Fourier-transform infrared spectroscopy (FTIR), scanning electron microscopy (SEM), and X-ray diffraction (XRD). Antibacterial properties were assessed against different microbial strains. In vitro antioxidant properties were evaluated through 2,2-diphenyl-1-picrylhydrazyl (DPPH) and 2,2-azino-bis-3-ethylbenzothiazoline-6-sulphonic acid (ABTS) assays. Bioactive compounds in the synthesized NPs were also identified.

Results: Analysis of the UV-vis spectrum confirmed the synthesis of ZnO NPs with an approximate size of 280 nm, as indicated by the absorption peak in the surface plasmon resonance band. FTIR spectroscopy revealed the presence of functional groups such as hydroxyl and carboxyl groups. SEM analysis determined the dimensions of the NPs to be around 11 nm. XRD patterns exhibited distinct Bragg reflections, confirming specific crystallographic planes. In vitro antioxidant assays demonstrated a reduction in absorbance at 517 nm and 734 nm, indicating antioxidant activity. Antibacterial testing revealed inhibition zones against *Escherichia coli*,* Staphylococcus aureus*,* Streptococcus mutans*,and *Enterococcus faecalis*.

Conclusion: The study successfully synthesized ZnO NPs using an eco-friendly method with garlic plant extracts. Characterization techniques confirmed the structural and chemical properties of the NPs. The synthesized NPs exhibited antioxidant and antibacterial activities, showcasing their potential for various applications. The identification of bioactive compounds further contributes to the understanding of the biological properties of the synthesized NPs.

## Introduction

Garlic (*Allium sativum *L.), a bulbous plant belonging to the Liliaceae family, is originally indigenous to Central Asia. However, it has established itself in numerous countries worldwide [1]. A wide array of metal and metal oxide nanoparticles (NPs) have been documented as being generated by biological systems, including bacteria, fungi, actinomycetes, yeasts, viruses, and plants [2]. The healthcare and food industries are confronted with a substantial challenge in the form of microbial contamination. As a result, there has been a growing emphasis on the exploration of antimicrobial agents and surface coatings in recent times [3]. The antioxidative capacity of mature extracts from all varieties has been evaluated through two separate spectrophotometric tests: the 2,2-diphenyl-1-picrylhydrazyl (DPPH) assay and the ferric reducing/antioxidant power (FRAP) assay [4]. However, as far as our understanding goes, there has been no previous investigation into the comparative antimicrobial effects of zinc oxide (ZnO) NPs synthesized through extracts from ginger (*Zingiber officinale*) and garlic (*Allium sativum*) nor has there been an exploration of the potential synergistic impact of a blend of these two extracts. Thus, the objective of this study was to evaluate and contrast the antibacterial properties of ZnO NPs produced using extracts from *A. sativum *bulbs and their combined formulation against two standard pathogens: gram-positive and gram-negative bacteria [5]. Considering their diversity and sustainability, plants stand out as the most promising candidates among other viable raw materials for the green synthesis of NPs [6]. In recent times, there has been a notable surge in the interest surrounding ZnO NPs as a promising avenue across various industries including optics, electronics, packaged foods, and medicine. This heightened attention primarily stems from their biocompatibility, minimal cytotoxicity, and cost-efficiency. Multiple research endeavors have underscored the pivotal role of zinc ions in triggering cell apoptosis by facilitating the production of reactive oxygen species (ROSs) and the release of potentially harmful zinc ions (Zn2+), which could pose risks to cells. Nonetheless, the practical application of ZnO NPs in clinical settings is impeded by the toxicity associated with the chemicals employed in their synthesis [7]. The advancement of nanoscience and nanotechnology is propelled by the distinct attributes of nanomaterials. Within this realm, metal oxide NPs have captivated substantial interest within the scientific domain. Their size-adjustable features confer a range of exceptional qualities, encompassing elevated chemical stability, a notable electrochemical coupling coefficient, an extensive radiation absorption spectrum, exceptional photostability, ready accessibility, affordability, and lack of toxicity. These characteristics render metal oxide NPs immensely attractive for diverse applications [8]. An array of diseases induced by microorganisms prevails on our planet. Hence, there arises a necessity to amass scientific knowledge concerning antibacterial agents that hold potential for incorporation into polyhydroxyalkanoate (PHA) materials, thereby furnishing a combination of biological and long-lasting antimicrobial safeguarding [9]. This study focuses on the ZnO NPs synthesized through aqueous extraction, employing advanced analytical techniques including a UV-visible (UV-vis) spectrophotometer, FTIR, SEM, and XRD and investigating their in vitro antibacterial properties. Additionally, the antibacterial property of the synthesized ZnO NPs was assessed.

## Materials and methods

Sample collection and preparation of *Allium sativum* extract for phytochemical analysis

The garlic powder applied in this experiment was procured from a nearby supplier in Chennai, Tamil Nadu, India, and was derived from coloration-dried plant materials. After gathering the plant materials, they were dried within the shade and were prepared for phytochemical analysis. To extract the phytochemicals, 500 g of dried *Allium sativum* leaf had been subjected to a Soxhlet extractor. The extraction procedure involved treating the pattern with 1250 ml of ethanol solvent for two hours. In the end, the resulting crude extract of *Allium sativum* was filtered using Whatman No. 41 clear out paper. The filtrate obtained was then evaporated at 60°C, using a rotary evaporator to yield a focused pattern. This focused pattern was later stored for future analysis to make sure its protection. The gathered crude *Allium sativum* ethanol extract was retained for future evaluation.

In vitro antioxidant activity

2,2-Diphenyl-1-Picrylhydrazyl (DPPH) Assay

The organic radical activity assay utilized the DPPH method with slight modifications. A 0.1 mM DPPH solution was prepared using methanol, and then, 100 µL of this solution was combined with 900 µL of eosinophil (EO) at varying concentrations ranging from 0 to 1000 µg/mL. The reaction tubes were then incubated at room temperature for 30 minutes while being shielded from the light. Subsequently, the absorbance was measured at 517 nm using a Genesys 20 spectrophotometer (Thermo Scientific, Waltham, MA, USA). Trolox was employed as the antioxidant control, and the inhibitory concentration (IC_^50^_) was deduced from the inhibition percentage plotted against the sample concentration [10].

ABTS Radical Scavenging Ability

The 2,2-azino-bis-3-ethylbenzothiazoline-6-sulphonic acid (ABTS) radical scavenging capacity was evaluated following a previously described procedure with minor adjustments. In essence, equal volumes of ABTS solution (7 mmol/L) and K2SO4 solution (2.45 mmol/L) were blended and placed in a dark environment at 25°C for 16 hours. The mixture's absorbance at 734 nm was adjusted to 0.70 ± 0.01 by adding PBS, after which it was combined with the fermented juice in equivalent amounts and maintained at 25°C for six minutes. The UV-vis spectrophotometer was employed to measure the absorbance of each sample at 734 nm. The blank control consisted of distilled water, and the calculation of the ABTS radical scavenging capacity was performed using the provided equation [11].

ABTS radical scavenging ability (%) = (A blank - A sample)/A blank × 100

Synthesis of ZnO NPs

The synthesis of ZnO NPs commenced by boiling the final aqueous extract on a magnetic stirrer, followed by the addition of 5 mL of a 10 mM Zn(NO3)2•6H2O solution. The solution displayed a faint yellow hue, which grew more pronounced during heating at 65°C over 40 minutes. This temperature was established as optimal after exploring various temperature settings. At 60°C, the discernible yellow color indicating the formation of zinc complexes with phenolic ligands was absent, and at 70°C, ash formation occurred. Consequently, 65°C was determined to be the ideal temperature for the study. The heating process triggered a transformation from zinc nitrate to zinc complexes, evident from the solution's yellow tint. Subsequently, the solution was dried by being heated at 65°C overnight, resulting in a dense paste. This paste was further subjected to comprehensive drying through calcination at 400°C for two hours. Calcination, a temperature-dependent process, facilitates the conversion of compounds to their impurity-free powdered state. Additionally, it enhances the crystalline nature of NPs, influencing their size and morphology [12].

Characterization of UV-Vis Spectrophotometry, FT‑IR, SEM, and XRD NPs

The surface plasmon resonance (SPR) of the silver (Ag) NPs synthesized via environmentally friendly methods was tested using a UV-vis spectrophotometer (Medical UviLine 9400C, Thermo Fisher Scientific, Loughborough, United Kingdom). For the evaluation of practical businesses and floor chemistry of the dried *A. sativum* extract derived from Citrus lemon zest and the synthesized AgNPs, FTIR spectroscopy was performed using an Agilent Cary 640 FTIR spectrometer. The spectra have been recorded at room temperature across a variety of 400-4000 cm^1^. Additionally, the particle size and dynamic light scattering of the AgNPs were ascertained using a Zetasizer Nano tool (Malvern) at a temperature of 25°C and a detection perspective of 90 ranges. The morphology and elemental composition of the AgNPs have been explored using a JEOL JSM-5910 SEM. The SEM evaluation was completed at an acceleration voltage of 20 kV, with a picture size resolution of ×30,000. XRD measurements had been carried out using a Philips (PW 1710) diffractometer running at 40 kV and 40 mA, with Cu(Kα) radiation (λ = 1.5406 Å), protecting a diffraction perspective (2θ) variety of 10° to 90°.

ZnO NP Synthesis of Antibacterial Allium sativum Aqueous Extract

The antibacterial activity of ZnO NPs was tested against (a) *Escherichia coli*, (b) *Staphylococcus aureus*, (c) *Streptococcus mutans*, and (d) *Enterococcus faecalis* by well-diffusion method. Three wells were made within the agar plate and were loaded with 20 µl of ZnO NPs (1 µg/µl), distilled water (terrible manipulate), and ZnSO4 (1 µg/µl). Antibiotic disc levofloxacin became used as wonderful control. The plates have been incubated for 24 h at 35°C. The antibacterial activity of ZnO NPs on the tested bacterial line was decided by using the formation of inhibition region across the wells.

Statistical analysis

The statistical analysis in this study involved the utilization of tools such as performing a one-way analysis of variance (ANOVA) to evaluate the significance of disparities among the diverse treatment groups. For pinpointing unique differences amidst the numerous treatment groups, Duncan's multiple range test (DMRT), a couple of variety checks, was achieved using the IBM SPSS Statistics for Windows, Version 16 (Released 2007; IBM Corp., Armonk, New York, United States). Effects have been taken into consideration statistically substantial while the p-value become much less than 0.05 (p < 0.05).

## Results

Phytochemical analysis

**Figure 1:**
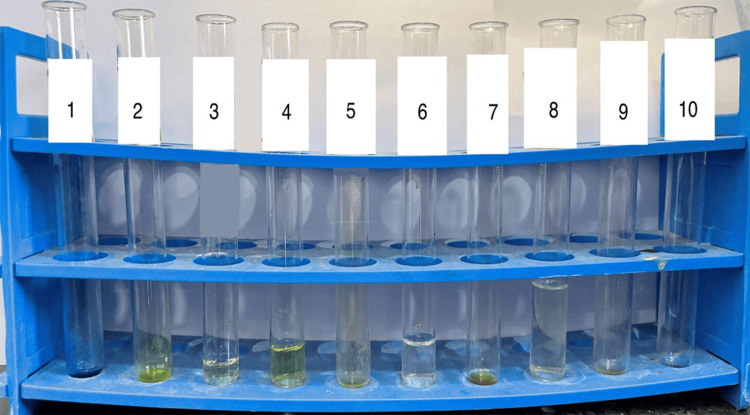
Secondary metabolite analysis of the Allium sativum aqueous extract

The phytochemical analysis of *A. sativum* aqueous extraction showed the presence of alkaloids, reducing sugars, glycosides, proteins and amino acids, phenolic compounds, flavonoids, tannin, and quinone and the absence of reducing sugars (Figure 1, Table 1).

**Table 1:** Phytochemical analysis of the Allium sativum aqueous extract

S. no	Secondary metabolites	*Allium sativum* aqueous extract
1	Reducing sugars (Fehling’s test)	-
2	Alkaloids	+
3	Reducing sugars (aqueous NaOH test)	+
4	Glycosides	+
5	Proteins and amino acids	+
6	Phenolic compounds	+
7	Flavonoids	+
8	Tannin	+
9	Quinone	+

In vitro antioxidant property of DPPH and ABTS assay

**Figure 2:**
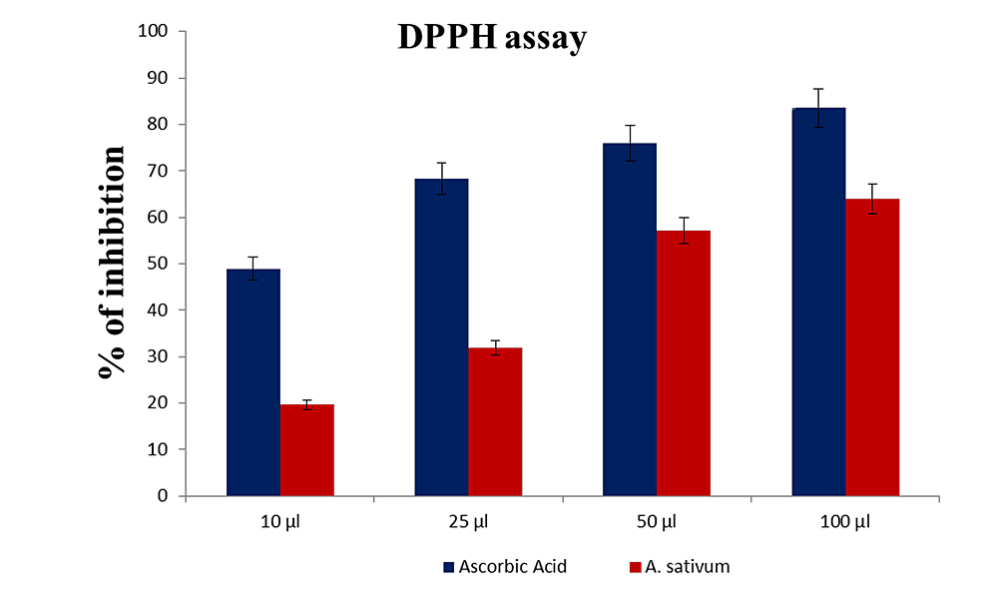
In vitro antioxidant property of DPPH DPPH:  2,2-diphenyl-1-picrylhydrazyl

**Figure 3:**
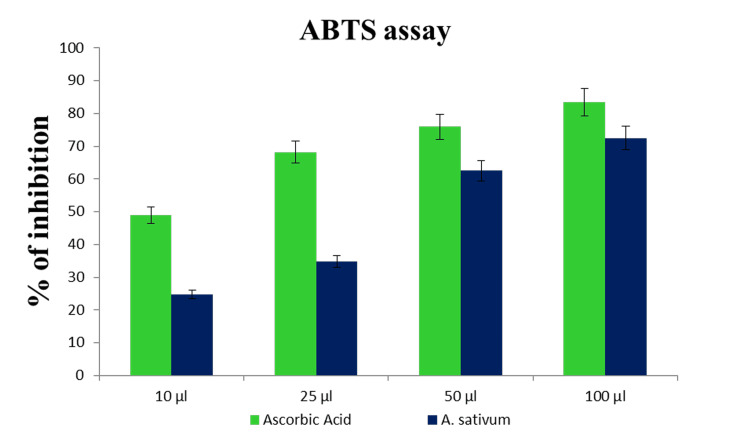
In vitro antioxidant property of ABTS ABTS: 2,2-azino-bis-3-ethylbenzothiazoline-6-sulphonic acid

The effects of in vitro antioxidant properties were evaluated through DPPH, and it was found that diallyl disulfide (44.21%) is the predominant thing of the vital oil (EO), observed by using diallyl disulfide (22.08%), allyl methyl disulfide (9.72%), 2-vinyl-4H-1,three-dithiine (4.78%), and α-bisabolol (3.32%) (Figures 2, 3).

Fermentation generates bioactive ingredients that effectively counteract the accumulation of free radicals. To assess the antioxidant capacity of fermented juice in vitro, ABTS, DPPH, and O2 assays are commonly utilized. These assays help measure the juice's ability to scavenge free radicals.

Characterization of UV and FTIR *Allium sativum *aqueous extract

**Figure 4:**
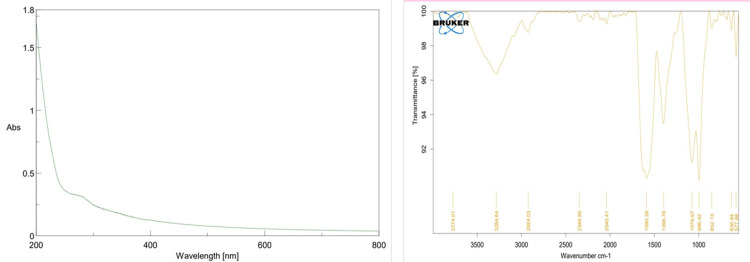
(a,b) Characterization of UV-visible ZnO nanoparticles ZnO: Zinc oxide

UV-vis spectroscopy was used for the analysis of ZnO NP-synthesized *Allium sativum* aqueous extract of high peak UV-Vis absorption spectra of 280 nm (Figure 4a, 4b).

FTIR spectroscopy and Raman spectra analysis of the functional group of Raman spectroscopy proved to be effective in identifying that the presence of cadmium selenide (CdSe) and reduced graphene oxide (RGO) in the composites is evident. The Raman spectra of the CdSe/RGO-1:2 complexes are displayed along the ones of natural CdSe. The Raman spectra display awesome longitudinal optical (LO) vibration peaks of CdSe at 203 cm^−1^ and 409 cm^−1^, corresponding to the 1LO and 2LO peaks, respectively (Figure 6).

Synthesis of SEM and XRD* Allium sativum* aqueous extract

**Figure 5:**
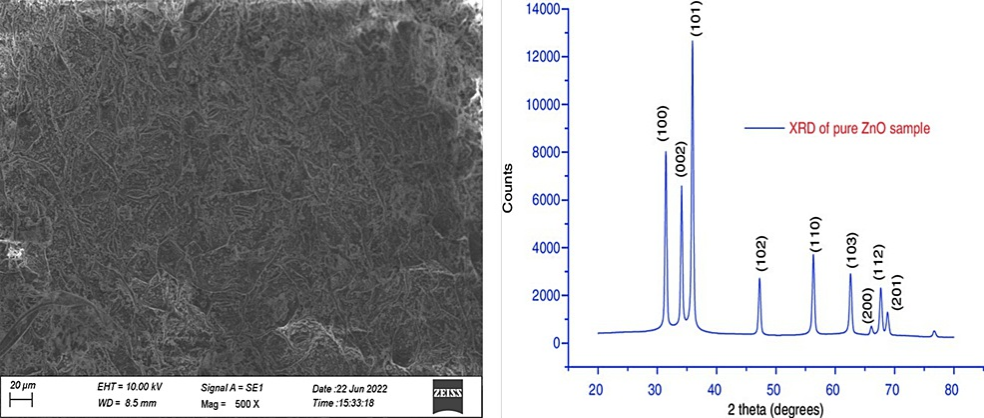
(a,b) SEM and XRD characterization of ZnO NP synthesis ZnO NP: Zinc oxide nanoparticle

SEM analysis determined that the dimensions of the AgNPs were approximately 11 nm (Figure 5a, 5b).

The composite adsorbent oyster shell (OS) exhibits irregular sheet-layered architectures with a porous structure designed to accommodate magnetic NPs. In the XRD evaluation of the CdSe/RGO-1:2, CdSe/RGO-1:1.5, CdSe/RGO-1:1, and CdSe/RGO-1:0.5 samples, the top function of graphene oxide at 9.7 tiers vanished upon the attachment of CdSe to graphene.

ZnO NP synthesis of the antibacterial properties of the *Allium sativum* aqueous extract

**Figure 6:**
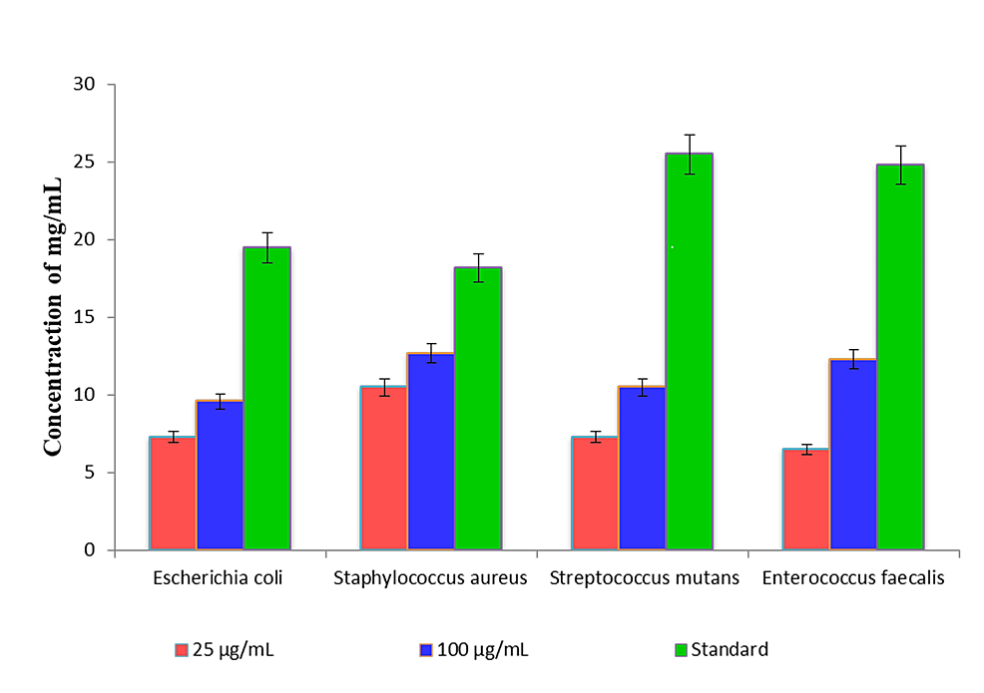
Antibacterial activity of ZnO NPs synthesized from the aqueous extract of Allium sativum

**Figure 7:**
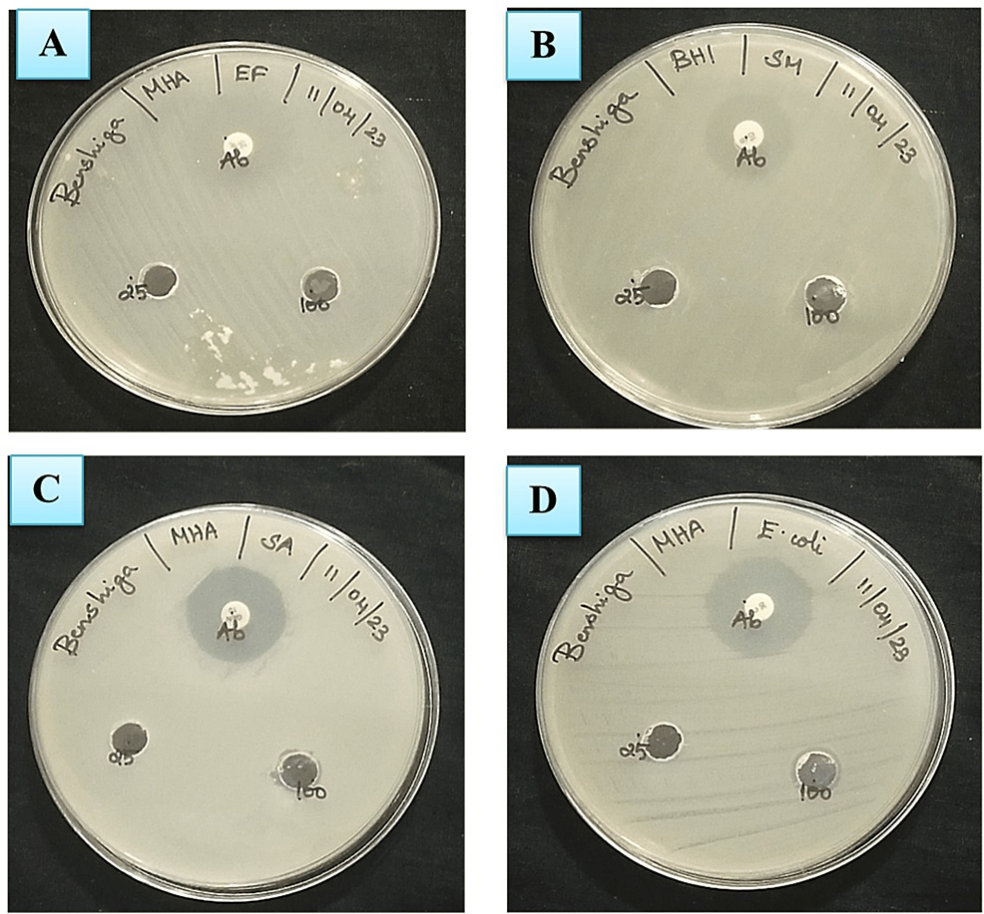
(a,b) Characterization of ZnO NP synthesis of the antibacterial Allium sativum aqueous extract ZnO NP: Zinc oxide nanoparticle

The ZnO NP synthesis derived from the *Allium sativum* leaf extract exhibited a sizeable quarter of inhibition against *P. aeruginosa* (20.3 mm) and *E.* *coli *(19.8 mm), whereas the zone of inhibition (Figure 6) was observed less for* Bacillus subtilis *(8.1 mm) and *S. aureus* (10.7 mm) (Figure 7).

 

## Discussion

Phytochemicals provide *Zingiber officinale* (ginger) roots with their color, flavor, and aroma while also serving as a crucial component of the plant's natural defense system. These compounds protect the plant against herbivorous insects, vertebrates, fungi, pathogens, and parasites [13]. The findings suggest that all SSF samples exhibited a large scavenging effect toward DPPH radicals in a dose-structured way across various concentrations [14]. At 5 mg/mL, the very best awareness tested for 50% jujube medium with *C. militaris* (CFJ) and control rice medium with 0% jujube (CFR), the scavenging ratios were determined to be 95.17% and 77.17%, respectively. Extensively, the DPPH hobby of CFJ at 5 mg/mL carefully approached that of the high-quality manipulated vitamin C (VC) (about 98.59%). Moreover, the water extracts of CFJ validated extensively better the DPPH radical scavenging outcomes than those of CFR. This observation suggests that the incorporation of jujube in the culture medium enhanced the antioxidant activity of solid-state fermentation (SSF)-jujube with *C. militaris *[15]. During these assays, a single electron from the free radicals combines with a radical scavenger present in the fermented juice. As a result, the violet color of the free radicals diminishes, leading to a reduction in absorbance at 734 nm. This decrease in absorbance indicates the successful neutralization of free radicals by the bioactive compounds found in the fermented juice [16]. The biosynthesized ZnO NPs showed maximum UV-vis spectroscopy absorbance at a wavelength of 360 nm [3]. The CdSe NPs inside the CdSe/RGO composite exhibited uniform dispersion on the graphene surface, as obtrusive from the FTIR and Raman spectra. This finding showed that the presence of oxygen-containing useful functions, along with hydroxyl (-OH) and carboxyl (-COOH) groups at the graphene surface, plays an important function in facilitating the dispersion of CdSe [17].

During the synthesis of this adsorbent, the modified NPs tend to aggregate and firmly adhere to both the surface and pore channels of OS. The encapsulation of hyaluronic acid (HA)-modified Fe3O4 significantly increases the number of active sites available [18]. The disappearance indicates the removal of oxygen-containing useful organizations, along with hydroxyl and carboxyl organizations, resulting in the reduction of graphene oxide [17]. Because of the excessive intensity of the CdSe diffraction height, the diffraction height band of RGO at 2θ = 26.6° couldn't be found. Additionally, the positions of the diffraction peaks within the composite cloth and CdSe had been equal at 2θ = 25.5°, 29.5°, 42.2°, and 49.9°. These four angles correspond to the 111, 200, 220, and 311 crystal planes of CdSe, respectively [Figure 6], indicating that the received CdSe NPs possessed a cubic zincblende shape [19].

Similar outcomes have been acquired regarding the antibacterial effect on *P. aeruginosa* and *E. coli *with the aid of synthesized ZnO NPs [20]. Studies have proven that increasing the concentrations of AgNPs significantly inhibits the increase of fungi [21]. Additionally, several research have tested extensive antifungal pastime of the synthesized AgNPs. To assess their antifungal capacity, the AgNPs have been tested in opposition to decide on pathogenic fungi, inclusive of *F. oxysporum, C. albicans, C. glabrata, *and *A. alternata*. Among these, the bottom antifungal hobby was located in opposition to *C. albicans*, accompanied by *C. glabrata *and *F. oxysporum* [22]. The findings of the study indicated the effectiveness of the AgNPs in opposition to all three fungal lines. *Fusarium graminearum* exhibited the highest inhibition area discovered, measuring 12.1 ± 0.01 mm at an attention of 80 µg/mL. Minimal inhibition zones of 9.8 ± 0.04 mm and 10.7 ± 0.03 mm were located against *Alternaria alternata *and *C. albicans,* respectively, additionally at an awareness of 80 µg/mL [12,23]. The synthesized AgNPs confirmed amazing antimicrobial hobby toward *Staphylococcus aureus*, as evidenced by using a sector of inhibition measuring 16.8 ± 0.03 mm at an excessive attention of 80 µg/mL. The antimicrobial properties of AgNPs continue to be notably studied, with amoxicillin usually used as preferred.

In vivo studies are necessary to validate the observed antimicrobial efficacy, in addition to assessing the stableness, toxicity, and actual-international software efficacy of the synthesized NPs.

## Conclusions

Here, ZnO NPs were successfully organized through an inexperienced synthesis technique using aqueous extracts of garlic (*A. sativum*). The bioactivity of the green-synthesized ZnO NPs was assessed by analyzing their antioxidant and antibacterial activities and evaluating them with those of chemically synthesized ZnO NPs. UV-vis spectrum analysis confirmed the dimensions of the NPs at 280 nm absorption, discovered through the floor plasmon resonance band. The dimensions of AgNPs had been analyzed through SEM, revealing a length of 11 nm. Moreover, records indicated that all ZnO samples possessed a hexagonal quartzite shape. ZnO NPs tested effectiveness as antibacterial dealers against not unusual pathogenic microorganisms. It is believed that utilizing plant extracts inside the artificial method enhances the antibacterial and antioxidant residences of ZnO NPs. Our work confirms the potential use of selected plant life in pharmaceutical and scientific industries for the gain of society.
